# Supplementary material for: Proteomics of Two Thermotolerant Isolates of Trichoderma under High-Temperature Stress
Source: J Fungi (Basel). 2021 Nov 24;7(12):1002. doi: 10.3390/jof7121002 (PMC8704589; doi:10.3390/jof7121002)
Supplement: Supplementary file 1 [file jof-07-01002-s001.zip › Fig S1.pdf]

Supplementary Fig. 1. 3D view of differential intensity levels of protein spots identified in thermotolerant isolates of *Trichoderma* during the heat stress. Spot intensity was quantified by Image Master 2-D Platinum V6.0 image analysis software (GE Healthcare). The images show a peak for each protein spot, with a peak height that is proportional to the spot intensity. (C: control; 1 h: Heat stress at 48°C for 1 h; 4 h: Heat stress at 48°C for 4 h)

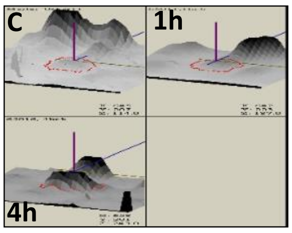

Spot: 352

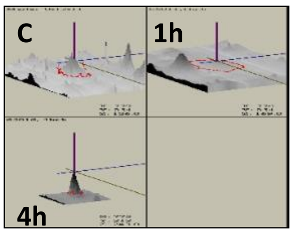

Spot: 445

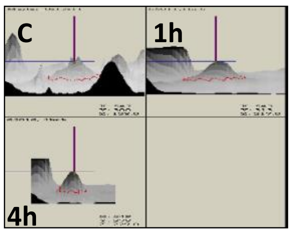

Spot: 528

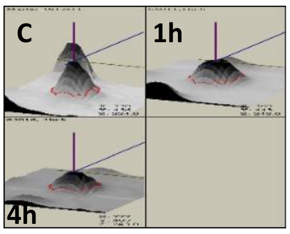

Spot: 732

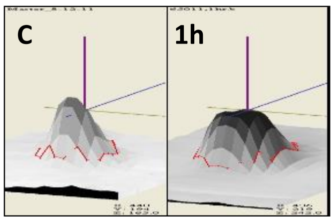

Spot: 329

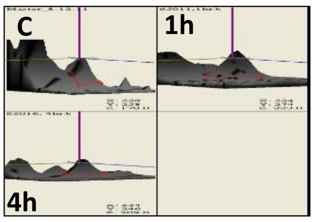

Spot: 450

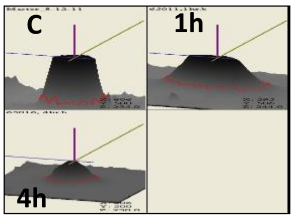

Spot: 817

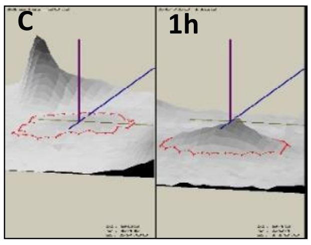

Spot: 904

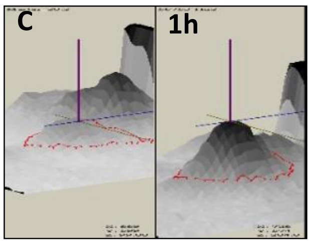

Spot: 856

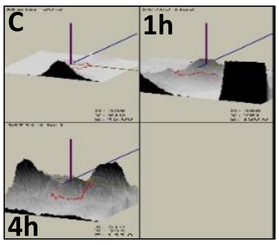

Spot: 1067

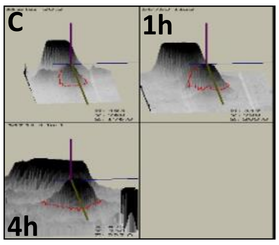

Spot: 1026

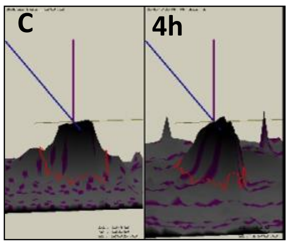

Spot: 1095

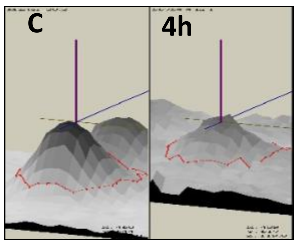

Spot: 901

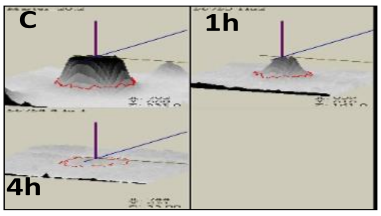

Spot: 920

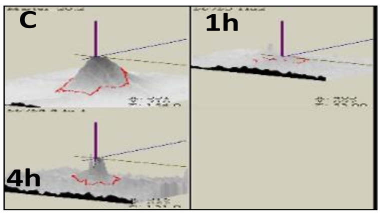

Spot: 984

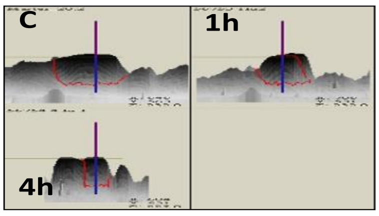

Spot: 794

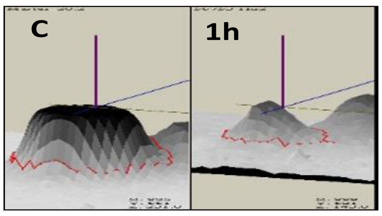

Spot: 922

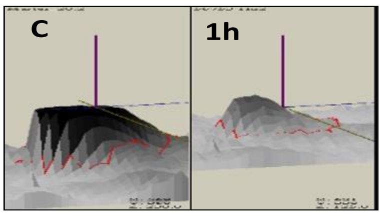

Spot: 485

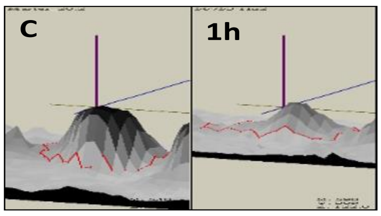

Spot: 446

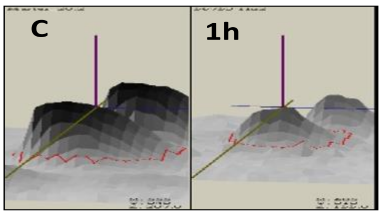

Spot: 452
