# Supplementary material for: Proteomics of Two Thermotolerant Isolates of Trichoderma under High-Temperature Stress
Source: J Fungi (Basel). 2021 Nov 24;7(12):1002. doi: 10.3390/jof7121002 (PMC8704589; doi:10.3390/jof7121002)
Supplement: Supplementary file 1 [file jof-07-01002-s001.zip › Table S1.pdf]

Table S1. List of accession numbers of thermotolerant *Trichoderma* isolates

**Isolate**	**Accession numbers**	
Gene	TEF1α	RNA Polymerase subunit B
T. asperellum 7316, TaDOR7316	KM190858	KX389492
T. longibrachiatum 673, TaDOR673	KM190859	KX389493
