# Supplementary material for: Proteomics of Two Thermotolerant Isolates of Trichoderma under High-Temperature Stress
Source: J Fungi (Basel). 2021 Nov 24;7(12):1002. doi: 10.3390/jof7121002 (PMC8704589; doi:10.3390/jof7121002)
Supplement: Supplementary file 1 [file jof-07-01002-s001.zip › Table S2.pdf]

Table S2. BLASTP and CDD (Conserved domain) analyses of protein identified during heat stress in *T. longibrachiatum* 673, TaDOR673

Expression Pattern	Spot no.	NCBI Hit of BlastP against *Trichoderma*	Fold-regulation		Conserved domains	Accession No.	Score	E-value	%Identity
			1 h	4 h					
Downregulated in 1 h and 4 h compared to control	871	hsp60 mitochondrial precursor-like protein [*Trichoderma reesei* QM6a]	-4.7	-16.48	GroEL domain	XP_006961679.1	140	2e-40	86%
	727	hypothetical protein TRIVIDRAFT_185457 [*Trichoderma virens* Gv29-8]	-1.07	-1.5	FES1 and ARM domain	EHK27263.1	143	2e-40	44%
	737	predicted protein [*Trichoderma reesei* QM6a]	-2.0	-3.9	Helicase domains	XP_006961979.1	716	0.0	49%
	553	glia maturation factor beta [*Trichoderma reesei* RUT C-30]	-4.0	-1.6	Actin depolymerization factor/cofilin-like domains (ADF domains)	ETS05399.1	95.9	1e-26	39%

Unique to control and absent in treated samples	CN2	hypothetical protein TRIATDRAFT_38099 [*Trichoderma atroviride* IMI 206040]	nd	nd	Phosphoadenosine phosphosulfate reductase family	EHK45271.1	184	4e-56	42%
Upregulated in 1 h compared to control and 4 h	329	phosphopyruvate hydratase [*Trichoderma reesei* QM6a]	4.43	nd	Enolase	XP_006963341.1	795	0.0	88%
Upregulated in 1 h and down regulated in 4 h compared to control	817	predicted protein [*Trichoderma reesei* QM6a]	1.95	-1.0	PWWP domain	XP_006966270.1	51.6	1e-06	26%
Upregulated in 4 h compared to control	576	hypothetical protein TRIVIDRAFT_181102 [*Trichoderma virens* Gv29-8]	nd	7.28	Oxidoreductase family	EHK20255.1	31.2	1.8	24%
	590	glycosyltransferase family 8 protein [*Trichoderma atroviride* IMI 206040]	nd	3.0	Glucosyl transferase 8 (GT8) family	EHK46197.1	105	1e-24	23%
	351	phosphopyruvate hydratase [*Trichoderma reesei* QM6a]	nd	4.05	Enolase superfamily	EHK50091.1	790	0.0	87%

Upregulated in 1 h and 4 h compared to control	450	predicted protein[*Trichoderma reesei* QM6a]	2.51	2.0	No conserved domains	XP_006962894.1	260	1e-86	100%
	352	hypothetical protein TRIVIDRAFT_30731 [*Trichoderma virens* Gv29-8]	1.2	10.2	Signal receiver domain	EHK24432.1	59.7	2e-09	29%
	445	heat shock factor-type DNA-binding domain-containing protein [*Trichoderma reesei* QM6a]	1.3	1.5	Heat shock transcription factor	XP_006965998.1	95.9	5e-20	27%
	528	Phosphopyruvate hydratase [*Trichoderma reesei* QM6a]	1.47	2.2	Enolase	XP_006963341.1	795	0.0	88%
	732	Glycosyltransferase family 4 protein [*Trichoderma atroviride* IMI 206040]	2.43	3.42	Glycosyltransferase group 1 family	EHK45433.1	233	4E-68	34%

nd: Not detected
1 h: Heat stress treatment at 48°C for 1 h
4 h: Heat stress treatment at 48°C for 4 h
