# Supplementary material for: Proteomics of Two Thermotolerant Isolates of Trichoderma under High-Temperature Stress
Source: J Fungi (Basel). 2021 Nov 24;7(12):1002. doi: 10.3390/jof7121002 (PMC8704589; doi:10.3390/jof7121002)
Supplement: Supplementary file 1 [file jof-07-01002-s001.zip › Table S3.pdf]

Table S3. BLASTP and CDD (Conserved domain) analyses of protein identified during heat stress in *T. asperellum* 7316, TaDOR7316

Expression Pattern	Spot no.	NCBI Hit of BlastP of protein against *Trichoderma*	Fold-regulation		Conserved domains	Accession No.	Score	E-value	%Identity
			1 h	4 h					
Unique to 1 h treated samples	1B	Intra-Golgi transport complex, subunit 6 [*Trichoderma reesei* QM6a]	-	nd	Conserved oligomeric complex (COG6)	XP_006965592.1	99.8	2e-21	21%
	1C	small heat shock protein [*Trichoderma harzianum*]	-	nd	Hsp20/alpha crystalline family	AAX55622.1	97.1	2e-24	37%
Downregulated in 1 h compared to control	485	hypothetical protein TRIATDRAFT_301560 [*Trichoderma atroviride* IMI 206040]	-2.9	nd	NAD(P)-binding Rossmann-like domain	EHK40773.1	855	0.0	77%
	446	DNA polymerase epsilon, catalytic subunit A [Trichoderma reesei QM6a]	-2.6	nd	DNA polymerase type-B epsilon subfamily catalytic domain	XP_006964819.1	2130	0.0	48%
	922	hypothetical protein TRIATDRAFT_87546	-4.3	nd	No conserved domains	EHK44512.1	22.3	4.8	86%

		[Trichoderma atroviride IMI 206040]							
	452	GTP cyclohydrolase [*Trichoderma reesei* QM6a]	-2.3	nd	GTP cyclohydrolase II (RibA)	XP_006967668.1	728	0.0	68%
Upregulated in 1 h and 4 h compared to control	1026	hypothetical protein TRIATDRAFT_52866 [*Trichoderma atroviride* IMI 206040]	2.2	4.2	tRNA threonylcarbamoyl adenosine modification protein YeaZ; Inactive homolog of metal-dependent proteases (COG1214); Glycoprotease family (Peptidase_M22)	EHK50571.1	498	2e-176	69%
	1067	hypothetical protein TRIATDRAFT_265826 [*Trichoderma atroviride* IMI 206040]	8.19	7.7	mRNA capping enzyme (pfam03291); SAM-dependent methyltransferases	EHK44240.1	494	4e-168	58%
Upregulated in 4 h compared to control and 1 h	1095	subtilisin like protease [*Trichoderma virens* Gv29-8]	nd	2.1	Peptidase S8 family domain in Protein convertases;	EHK25893.1	564	0.0	43%
Unique to control and absent in	C7	vacuolar sorting protein [*Trichoderma reesei* QM6a]	nd	nd	Non-viral sialidases; VPS10 domain	XP_006962518.1	1559	0.0	53%

treated samples	C8	Serine/threonine-protein phosphatase 2B catalytic subunit [*Trichoderma reesei* RUT C-30]	nd	nd	PP2B, metallophosphatase domain	ETS03163.1	828	0.0	78%
Down regulated in 4 h compared to control	901	predicted protein [*Trichoderma reesei* QM6a]	nd	-1.14	Mitochondrial PGP phosphatase	XP_006964630.1	181	2e-57	50%
Upregulated in 1 h compared to control	904	tripeptidyl-peptidase 1 precursor [*Trichoderma reesei* RUT C-30]	2.4	nd	Peptidase domain in the S53 family	ETR98149.1	377	4e-122	41%
	856	hypothetical protein TRIVIDRAFT_213745 [*Trichoderma virens* Gv29-8]	2.7	nd	chromosome segregation protein SMC; Autophagy protein Apg6	EHK19294.1	434	1e-143	45%
Down regulated in 1 h and 4 h compared to control	920	vacuolar serine protease [*Trichoderma atroviride*]	-4.18	-7.8	Peptidase S8 family domain	ABG57252.1	642	0.0	65%
	984	hypothetical protein TRIVIDRAFT_209259 [*Trichoderma virens* Gv29-8]; antiviral helicase [*Trichoderma reesei* RUT C-30]	-6.2	-3.7	DEAD-like helicases superfamily	EHK22776.1; ETS02258.1	1116; 427	0.0; 8e-130	49%; 41%

	794	fructose bisphosphate aldolase [*Trichoderma reesei* QM6a]	-2.1	-3.0	Class II Type A, Fructose-1,6-bisphosphate (FBP) aldolases	XP_006968688.1	661	0.0	91%

nd: Not detected
1 h: Heat stress treatment at 48°C for 1 h
4 h: Heat stress treatment at 48°C for 4 h
